# Determination of antioxidant and antimicrobial activities of leaf extracts of *Otostegia integrifolia*

**DOI:** 10.1186/s13065-018-0433-2

**Published:** 2018-05-18

**Authors:** Yiketel Adege Chekol, Zelalem Yibralign Desta

**Affiliations:** Department of Chemistry, College of Science, Bahir Dar University, P. O. Box 79, Bahir Dar, Ethiopia

**Keywords:** *Otostegia integrifolia*, Flavonoid content, Antioxidant activity, Antibacterial activity

## Abstract

**Background:**

The extracts from the leaves of *Otostegia integrifolia* have been reported to show phytochemical analysis, total flavonoid content, antioxidant and antibacterial activities.

**Results:**

Our results revealed that the total flavonoid content of methanol and ethyl acetate extracts is 416.5 + 0.288 and 248.9 + 0.872 mgAAE/100 g respectively. The two extracts also showed good antioxidant activity as well as weak to moderate antibacterial activity against some bacteria.

**Conclusions:**

The leaf extracts from *O. integrifolia* showed good total flavonoid content, DPPH radical scavenging activity and antibacterial activity. In addition to this, the extracts also showed the presence of some important compounds by phytochemical analysis.

## Background

*Otostegia integrifolia,* more commonly known as Abyssinian rose, a plant belonging to the family *Lamiaceae*, is endemic to Ethiopia, in the dry evergreen woodlands of the Tigray, Gondar, Wollo, Gojjam, North Shewa, Kaffa and Hararghe regions, as well as in the dry and moist agro climatic zones of the district known as Dega [1]. The plant is also endemic to Eritrea and Yemen [2]. *Otostegia integrifolia* is a shrub which grows up to 3 m tall, often with paired spines at the nodes. Its leaves are sessile or shortly petiolate. The blade is bluish greyish-green, oblanceolate to lanceolate shaped, and reaches 2–9 cm long [3, 4]. The plant grows in the wild but is also cultivated in gardens. It grows on mountain bush lands and wood lands over grazed slopes at altitudes ranging from 1300 to 2800 m. In Ethiopian traditional medicine, the leaves of *O. integrifolia* are used for the treatment of several diseases including malaria, for treatment of ophthalmia, as an anti-microbial, antihyperglycemic, and for its anti-oxidant properties used in preventing different kinds of sickness and disorders [5]. In this paper we reported phytochemical analysis, total flavonoid content, antioxidant and antibacterial activities of *O. integrifolia* leaf extracts.

## Results and discussion

The results of phytochemical analysis, total flavonoid content, antioxidant activity and antimicrobial activity tests obtained from different extracts of the leaves of *O. integrifolia* will be discussed as follows.

### Phytochemical analysis

This study showed the presence of different bioactive compounds in different solvent extracts of the leaves of *O. integrifolia* by using color change as a confirmatory test. Methanol extract was found to have a wide range of bioactive compounds including flavonoids, phenols, terpenoids, saponins, steroids and glycosides because of its high polarity. The ethyl acetate extract was also positive for flavonoids, phenols, terpenoids, saponins, steroids and glycosides since ethyl acetate has medium polarity. The petroleum ether being highly non-polar in nature and was able to extract very limited compounds such as steroids and glycosides. Alkaloids and tannins were however absent in all extracts of the leaf parts of the plant. The result of the test was summarized as follows in Table 1.

**Table 1:** Qualitative analysis of phytochemicals present in leaf extracts of *O. integrifolia*

Phytochemicals	ME	EA	PE
Alkaloids	−	−	−
Flavonoids	++	+	−
Phenols	+	+	−
Tannins	−	−	−
Steroids	+	+	+
Saponins	+	+	−
Terpinoids	+	++	−
Glycosides	++	++	+

(++) highly present, (+) present, (−) not present

*ME* methanol extracts, *EA* ethyl acetate extract, *PE* petroleum ether extract

### Determination of the total flavonoid content

The total flavonoid content of the extracts expressed as quercetin equivalent (mgQE) per dry sample (Table 2). As it was observed from the table, both methanol and ethyl acetate extracts of the leaves of *O. integrifolia* were contain flavonoids. The total flavonoid content of methanol extract was 416.5 + 0.288 mgQE/100 g dry sample while that of ethyl acetate extract was 248.9 + 0.872 mgQE/100 g dry sample which mean methanol extract contains more flavonoids compared to ethyl acetate extract.

**Table 2:** Total flavonoid content of leaf extracts of *O. integrifolia*

Extracts	Absorbance at 510	mgQE/100 g of dry weight
Methanol extract	0.148 ± 0.0009	416.5 ± 0.288
Ethyl acetate extract	0.092 ± 0.0029	248.9 ± 0.872

Data expressed as mean of three determinations ± standard deviation

### Antioxidant activity

The antioxidant activities of the extracts of the leaves of *O. integrifolia* were evaluated by using FRAP and DPPH assays.

### Ferric reducing antioxidant power (FRAP) assay

In this method, ascorbic acid was used as a standard to determine antioxidant activities of the extracts of the leaves of *O. integrifolia*. From Table 3, we observed that methanol extract has higher mgAAE/100 g dry weight i.e., 286.146 + 0.889 mgAAE/100 g dry weight than ethyl acetate extract i.e., 219.496 + 0.566 mgAAE/100 g dry weight, that can strengthen the greater reducing power of methanol extract. The result of our study for each extract was supported by previous reported data by Anwar et al. [6] and this report revealed that the extracts of more polar solvents exhibited better antioxidant activities than that of less polar solvents.

**Table 3:** FRAP values of leaf extracts of *O. integrifolia* (mgAAE/100 g)

Extracts	FRAP value in mg AAE/100 g dry wt
Methanol extract	286.146 ± 0.889
Ethyl acetate extract	219.496 ± 0.566

### DPPH radical scavenging activity

The DPPH free radical scavenging ability of the extracts of the leaves of *O. integrifolia* was expressed using mgAAE/100 g of dry sample. The DPPH free radical scavenging ability of methanol and ethyl acetate extracts were evaluated by using color change as the reagent was added and recorded the absorbance of each extracts at different concentrations. The change of a color from pink to yellow in each extracts as well as standard solution confirmed that they have DPPH radical scavenging capacity. The faster the disappearance of the color revealed that the extract has higher DPPH free radical scavenging activity. According to our study, methanol extract showed change of the color from pink to yellow faster than that of ethyl acetate extract, then methanol extract has greater DPPH radical scavenging power compared to that of ethyl acetate extract as shown in Table 4.

**Table 4:** DPPH radical scavenging values of leaf extracts of *O. integrifolia*

Extracts	DPPH scavenging value (mg AAE/100 g of dry weight)
Methanol extract	82.91 ± 0.365
Ethyl acetate extract	32.68 ± 1.545

### Antibacterial activity

Antibacterial activity of the extracts of the leaf of *O. integrifolia* was evaluated by using Agar well diffusion method. Five bacteria were used for the determination in which three of them were gram negative bacteria (*E. coli*, *S. typhi and K. pneumoniae*) whereas the remaining two were gram positive bacteria (*S. aurous and S. pyogens*). As shown in Table 5, methanol extract showed a significant antibacterial activity whereas there was no inhibition zone recorded in petroleum ether extract in all five bacteria. Ethyl acetate extract had also a potential antibacterial activity against all five bacteria except *S. aurous*. All extracts had lowest antibacterial potential as compared to standards (*Gentamycin* and *Chloramphenicol*). In this study a good antibacterial activities were recorded using methanol extract compared to other extracts. For instance, methanol extract showed a good antibacterial result against *S. aureus*, *E. coli* and *S. typhi* with minimum zone of inhibition 13.5 + 0.40, 13.9 + 0.16 and 10.1 + 0.04 respectively however, the highest minimum inhibition zone was recorded in ethyl acetate extract against *S. pyogens* and *K. pneumoniae* with minimum zone of inhibition 17.1 + 0.14 and 16.8 + 0.41 respectively.

**Table 5:** Comparison of MZI among leaf extracts of *O. integrifolia*

Extracts and standard antibiotics	Concentration in µg/mL	Average values of zone of inhibition				
		*S. aurous*	*S. pyogens*	*E. coli*	*S. typhi*	*K. pneumoniae*
Methanol extract	25	11.4 ± 0.29	14.6 ± 0.25	8.5 ± 0.09	9..3 ± 0.03	6.4 ± 0.21
	50	11.7 ± 0.37	15.0 ± 0.36	9.7 ±0.59	9.9 ±0.16	7.6 ± 0.29
	75	12.2 ± 0.33	15.3 ± 0.33	11.1 ± 0.37	10.1 ± 0.04	10.3 ± 0.43
	100	13.5 ± 0.40	15.7 ± 0.26	13.9 ± 0.16	10.1 ±0.38	12.2 ± 1.03
Ethyl acetate extract	25	0	10.4 ±0.17	6.6 ± 0.12	6.4 ±0.05	12.6 ± 0.24
	50	0	11.6 ± 0.34	7.1 ± 0.17	6.9 ± 0.16	13.4 ± 0.34
	75	0	15.5 ± 0.1	7.8 ± 0.29	8.6 ±0.28	14.8 ± 0.26
	100	0	17.1 ± 0.14	8.6 ± 0.21	9.4 ± 0.49	16.8 ± 0.41
Petroleum ether extract	25	0	0	0	0	0
	50	0	0	0	0	0
	75	0	0	0	0	0
	100	0	0	0	0	0
Standard antibiotics	Gen.	24.5 ± 0.12	27.7 ± 0.08	25.0 ± 0.12	23.4 ±0.37	23.1 ±0.33
	Chl.	18.8 ± 0.21	31.4 ± 0.15	16.6 ± 0.17	15.5 ± 0.21	19.8 ±0.13

*S. aureus, Staphylococcus aureus*; *S. pyogens*, *Streptococcus pyogens*; *E. coli*, *Escherichia coli*; *S. typhi*, *Salmonella typhi*; *K. pneumoniae*, *Klebsiella Pneumoniae*; *Gen*, *Gentamycin*; *Chl*, *Chloramphenicol*

## Experimental section

### Chemicals and reagents

Ferric chloride (FeCl_3_), Wagner’s reagent (Iodine in potassium iodide), hydrated aluminum chloride (AlCl_3_.6H_2_O), sodium nitrite (NaNO_2_), hydrochloric acid, sulfuric acid (H_2_SO_4_), sodium hydroxide (NaOH), nitric acid (HNO_3_), sodium carbonate (NaCO_3_), monosodium hydrogen phosphate (NaH_2_PO_4_), disodium hydrogen phosphate (Na_2_HPO_4_), trichloroacetic acid, potassium hexacyanoferrate (II) (K_2_[Fe(CN)_6_], Ascorbic acid, 2,2-diphenyl-1-picrylhydrazyl (DPPH), quarticien, ammonia solution, chloroform, acetone, iodine powder, potassium iodide, Muller Hinton agar, ethyl acetate, methanol, petroleum ether, distilled water and deionized water were some of the chemicals and reagents that were used for the experimental work during our study.

### Plant materials

Fresh leaves of *O. integrifolia* were collected from Arebaya, which is located in north Gonder zone and 288 km away from Bahir Dar, Amhara regional state, Ethiopia in May 2017. The plant material was identified and authenticated by Dr. Ali Seidu, botanist in biology department, Bahir Dar University.

### Extraction of samples

The air-dried and ground (100 g) of the leaves of *O. integrifolia* were extracted by soaking successively in *n*-hexane, ethyl acetate (EtOAc) and methanol (MeOH) each for 24 h (two times with each solvent) and removal of the solvent under reduced pressure using a BUCHI flash evaporator to afford extracts of 2.1 g (for *n*-hexane), 13.0 g (for EtOAc) and 18.5 g (for MeOH).

### Phytochemical analysis

The phytochemical analysis of methanol, ethyl acetate and petroleum ether extracts of the leaves of *O. integrifolia* were studied by slight modifications based on standard procedures described on different literatures [7–10].

### Measurement of total flavonoid content

Total flavonoid content was measured with aluminum chloride colorimetric assay as described by different researchers with minor modifications [10, 11]. In brief, 1 mL of methanol and ethyl acetate extracts and 1 mL of standard quercetin solutions (20, 40, 60, 80 μg/mL) were positioned into test tubes and 4 mL of distilled water and 0.3 mL of 5% sodium nitrite solution were added into each solutions. After 5 min, 0.3 mL of 10% aluminum chloride was added. At 6th min, 2 mL of 1 M sodium hydroxide was added and orange yellowish color was developed. The absorbance was measured at 510 nm by using UV–visible spectrophotometer. The blank was performed using distilled water. Quercetin was used as standard. The samples were performed in triplicates. The calibration curve was plotted using standard quercetin. The data of the total flavonoid contents was expressed as mg of quercetin equivalents/100 g of dry mass.

### Measurement of free radical scavenging activity

#### DPPH radical scavenging assay

The antioxidant activity of methanol and ethyl acetate extracts was measured on the basis of the scavenging activity of the stable 1,1-diphenyl-2-picrylhyorazyl (DPPH) free radical according to the method described by Thaiponga et al. with slight modifications [10, 12, 13]. In brief, 1 mL of DPPH solution was added to 4 mL of various concentrations of methanol and ethyl acetate extracts and ascorbic acid to be tested. After 30 min, absorbance was measured at 517 nm. Ascorbic acid with a series of concentration was used as a reference material. All tests were performed in triplicate.

#### Ferric reducing antioxidant power (FRAP) assay

The reducing power of methanol and ethyl acetate extracts was determined according to the method described by Abebe et al. [13] with slight modification [13]. In brief, 2.5 mL of different concentration of methanol and ethyl acetate extracts were mixed with 2.5 mL of phosphate buffer solution (PH = 6.6, 0.2 M) and 2.5 mL of potassium hexacyanoferrate ([K_3_Fe(CN)_6_]) (1%). The mixture was incubated at 50 °C for 20 min in water bath. Then 2.5 mL of Trichloroacetic acid (10%) was added to the mixture to terminate the reaction. 5 mL of the upper layer of the solution was mixed with 5 mL of distilled water and 0.5 mL of FeCl_3_ solution (0.1%). The reaction mixture was leave for 10 min at room temperature and the absorbance developed bluish green color was measured at 700 nm by using UV-spectrophotometer against a blank solution. Distilled water was used instead of extracts or standard to prepare a blank solution.

#### Antimicrobial activity

Antimicrobial activities were performed in microbiology laboratory, department of Biology, Bahir Dar University by using agar well diffusion method. Muller Hinton agar media was prepared for culturing selected gram negative and gram positive bacteria by using standard methods. Five bacteria [two gram positive (*S. aureus and S. pyogens*) and three gram negative (*E. coli, S. typhi and K. pneumoniae*)] were selected and collected from department of Biology, Bahir Dar University. A series of plant extract concentrations (25, 50, 75,100 μg/mL) and standard antibiotics (*Gentamycin* and *Chloramphenicol*) were added to the incubated plate by using filter paper. Then it was incubated for 24 h at 37 °C and the experiment was repeated three times, and average values of zone of inhibition was recorded in mm for antimicrobial activity as described before [14, 15].

#### Data analysis

The results were reported as mean ± standard deviation (SD). The calibration curves were constructed by using Microsoft excel window 10 and origin 8.

## Conclusions

In conclusion, we found that the leaf extracts from *O. integrifolia* showed good total flavonoid content, good DPPH radical scavenging activity and weak to moderate antibacterial activity. Among those extracts, methanol extract is the one that showed good activities compared to that of ethyl acetate and petroleum ether extracts.
